# Phosphatidyl-Inositol-3 Kinase Inhibitors Regulate Peptidoglycan-Induced Myeloid Leukocyte Recruitment, Inflammation, and Neurotoxicity in Mouse Brain

**DOI:** 10.3389/fimmu.2018.00770

**Published:** 2018-04-17

**Authors:** Daniela S. Arroyo, Emilia A. Gaviglio, Javier M. Peralta Ramos, Claudio Bussi, Maria P. Avalos, Liliana M. Cancela, Pablo Iribarren

**Affiliations:** ^1^Centro de Investigaciones en Bioquímica Clínica e Inmunología (CIBICI-CONICET), Departamento de Bioquímica Clínica, Facultad de Ciencias Químicas, Universidad Nacional de Córdoba, Córdoba, Argentina; ^2^Departamento de Farmacología (IFEC-CONICET), Facultad de Ciencias Químicas, Universidad Nacional de Córdoba, Córdoba, Argentina

**Keywords:** myeloid cells, phosphatidyl-inositol-3 kinase inhibitors, autophagy, peptidoglycan, TLR2, inflammation

## Abstract

Acute brain injury leads to the recruitment and activation of immune cells including resident microglia and infiltrating peripheral myeloid cells (MC), which contribute to the inflammatory response involved in neuronal damage. We previously reported that TLR2 stimulation by peptidoglycan (PGN) from *Staphylococcus aureus, in vitro* and *in vivo*, induced microglial cell activation followed by autophagy induction. In this report, we evaluated if phosphatidyl-inositol-3 kinase (PI3K) pharmacological inhibitors LY294200 and 3-methyladenine (3-MA) can modulate the innate immune response to PGN in the central nervous system. We found that injection of PGN into the mouse brain parenchyma (caudate putamen) triggered an inflammatory reaction, which involved activation of microglial cells, recruitment of infiltrating MC to injection site, production of pro-inflammatory mediators, and neuronal injury. In addition, we observed the accumulation of LC3B^+^ CD45^+^ cells and colocalization of LC3B and lysosomal-associated membrane protein 1 in brain cells. Besides, we found that pharmacological inhibitors of PI3K, including the classical autophagy inhibitor 3-MA, reduced the recruitment of MC, microglial cell activation, and neurotoxicity induced by brain PGN injection. Collectively, our results suggest that PI3K pathways and autophagic response may participate in the PGN-induced microglial activation and MC recruitment to the brain. Thus, inhibition of these pathways could be therapeutically targeted to control acute brain inflammatory conditions.

## Introduction

Regulation of immune response in the brain is in part accomplished by myeloid cells (MC), which represent a diverse group of mononuclear phagocytic cells (1–3). The healthy central nervous system (CNS) parenchyma contains only one type of MC, the parenchymal microglial cell, which are tissue-resident macrophage confined in an immunosuppressive environment (4, 5). However, during parenchymal brain injury, microglial cells become activated and mediate the local immune response. These cells are a prominent source of pro-inflammatory factors and oxidative stress mediators, such as tumor necrosis factor (TNF)-α, interleukin (IL)-1β, chemokines, nitric oxide (NO), all of which are further neurotoxic (6, 7). In response to tissue damage, infiltrated blood-borne MC migrate to the affected area and may contribute the neuroinflammatory process (8). Nevertheless, the differential roles of these MC populations in CNS disorders have only recently been acknowledged.

Toll-like receptors (TLRs) are a family of pattern-recognition receptors in the innate immune system. Exogenous and endogenous TLR ligands activate microglia that trigger inflammatory reactions in CNS (9–11). Recent studies using a mouse experimental brain abscess model have revealed a complex role for TLRs in the disease pathogenesis (12). Interestingly, TLR2 participates in the innate immune response during the acute stage of brain abscess formation induced by *Staphylococcus aureus* and influences adaptive immune response (13, 14). Moreover, MyD88, a central adapter molecule for many TLRs including TLR2, is a key component in the brain innate immunity and was involved in exaggerated brain tissue destruction (15, 16). Numerous reports suggest that TLR2 contributes to the phagocytosis and autophagy in professional phagocytes upon bacterial infection (17–19). In line with such evidences, we previously demonstrated that intracerebral delivery of peptidoglycan (PGN), the major surface component of Gram-positive bacteria and a potent TLR2 agonist, leads to the autophagy activation in microglial cells *in vivo* (20). In addition, we observed that both, 3-methyladenine (3-MA) and LY294002 inhibited autophagy activation in microglial cells and reduced NO production.

Autophagy is a conserved process, whereby cells deliver cytoplasmic contents to the lysosomes for removal (21). Although this mechanism has a main protective function (22), under several conditions, deregulated autophagy may contribute to the inflammation and tissue injury (23). Enhanced autophagy has been implicated in various neurological conditions including intracerebral hemorrhage, cerebral ischemia, and spinal cord injury (24, 25). This self-degradation process is emerging as a core regulator of CNS inflammation, aging, and neurodegeneration (26). In the brain, it has mostly been studied in neurons, where the delivery of toxic molecules and organelles to the lysosomes by autophagy is crucial for the neuronal health and survival (27, 28). The initiation and course of the autophagic flux are regulated by Beclin 1/Class III phosphatidyl-inositol-3 kinase (PI3K)-containing complexes (29). Nevertheless, PI3K have dual role in autophagy induction since, class III PI3K is required for the autophagosome formation but class I PI3K interact with principal negative regulator of autophagy mTOR (30).

PI3Ks regulate several key events in the inflammatory response to damage and infection and they were implicated in the regulation of the pro-inflammatory responses induced by TLR activation (31). However, the role of PI3Ks in the signaling pathways downstream of TLRs on MC is not completely clear. Different studies suggested that this pathway can play either positive or negative roles in the production of pro-inflammatory cytokines (32–34). Furthermore, most of the studies were performed in the peripheral immune system, and little is known about the contribution of the PI3K activity and autophagy in the regulation of the neuroinflammatory response elicited by PGN.

We propose that the inhibition of PI3K and autophagy could modulate CNS inflammation induced by TLR2 stimulation. Therefore, the aim of this study was to evaluate if intracerebral administration of PI3K inhibitors could regulate neuroinflammatory responses induced by PGN. We observed that this TLR2 ligand induced MC activation and colocalization of LC3B and lysosomal-associated membrane protein 1 (LAMP1) molecules in the CNS. In addition, PI3K inhibitors (including 3-MA) prevented the recruitment of inflammatory MC to the brain and reduced signs of neurodegeneration. Furthermore, here we show that PI3K inhibitors differentially regulated the PGN-elicited production of pro-inflammatory molecules and chemokine receptor expression in brain MC. Our findings suggest that induction of neuroinflammation by PGN was TLR2-dependent and it may require PI3K activation and autophagy. Inhibition of these pathways in the brain may lead to the downregulation of both, microglial cell activation and leukocyte recruitment to CNS, resulting in neuronal protection.

## Materials and Methods

### Reagents and Animals

Peptidoglycan from *S. aureus* and 3-MA were purchased from Sigma-Aldrich (St. Louis, MO, USA). LY294002 were purchased from Cell Signaling Technology, Inc. (Beverly, MA, USA). In this study, 6- to 8-week-old male C57BL/6J, TLR2 KO, or MyD88 KO mice were used. C57BL/6J mice were purchased from the Facultad de Ciencias Veterinarias, Universidad Nacional de La Plata, Argentina; C57BL/6 TLR2-knockout and C57BL/6 MyD88 knockout mice were purchased from The Jackson Laboratory, Bar Harbor, ME, USA. Animal care was provided in accordance with the procedures outlined in the “Guide for the Care and Use of Laboratory Animals” (NIH Publication No. 86-23, 1985). The experimental protocols were approved by the Institutional Animal Care and Use Committee of Centro de Investigaciones en Bioquímica Clínica e Inmunología (CIBICI), Consejo Nacional de Investigaciones Científicas y Técnicas (CONICET). Our animal facility obtained NIH animal welfare assurance (assurance number A5802-01, OLAW, NIH, USA).

### Surgical Procedures

After 1 week of acclimatization to the housing facility, 6- to 8-week-old male C57BL/6J mice were anesthetized with a combination of ketamine/xylazine. The mouse scalp was shaved and scrubbed with hydrogen peroxide. Animals were placed in a Thomas stereotaxic frame (Philadelphia, PA, USA). A midline incision was made, the skin was retracted, and one small bore hole was drilled into the skull. The infusion cannula (30 G; 20 mm) was stereotaxically lowered into the caudate putamen (CPU) using the following coordinates: anterior, +0.8 mm; lateral, +1.5 mm; ventral, −3.2.0 mm, according to the atlas of Franklin and Paxinos (2008). The infusion cannulae were connected *via* polyethylene tubing (PE 10; Becton Dickinson, Sparks, MD, USA) to 10 µl microsyringes (Hamilton, Reno, NV, USA) mounted on a microinfusion pump (Harvard Apparatus, Holliston, MA, USA). Each mouse was injected with 5 µg PGN alone or in combination with 0.19 nmol LY2942002 or with 6.37 nmol 3-MA at a volume 0.35 μl/side at a flow rate of 0.35 µl/min. This volume was selected according to the size and structure of these nuclei. Immediately after the microinjection, the cannulae were retracted, the holes were covered with wax, and the skin was sutured with surgical thread. At different time after surgery, mice were killed, and brains processed for analysis.

### FACS Analysis

For *ex vivo* analysis, at different time points, the mice brains were rapidly removed and placed on ice in an acrylic brain matrix (Stoelting Co., USA). Coronal brain slices of 2.0 mm containing the CPU from each hemisphere were dissected. The rest of brain tissues (BRAIN) were collected separately of CPU section, then all these sections were homogenized with scissors on ice and whole brain cells were costained with anti-CD45 (APC-Cγ7) anti-CD11b (PerCPe) antibodies, Ly6C (PE-Cγ7), IA/IE (APC), CD86 (PE), CX3CR1 (FITC), and CXCR4 (APC) (Becton Dickinson, San Jose, CA, USA). Stained cells were analyzed by flow cytometry on a FACSCanto II cytometer (Becton Dickinson, San Jose, CA, USA), using FACS DIVA™ software V 6.0, and data analysis was conducted using FCS express (*De Novo* Software).

Staining of single-cell suspension of isolated brain immune cells was performed using standard protocols. Briefly, cells were stained for surface markers for 30 min at 4°C and washed twice before analysis. All staining procedures were completed at 4°C in DPBS containing 5 mM EDTA and 1% FCS. For all assays, the frequency of resident myeloid cells (RMC) or microglial cell was determined by flow cytometry gating on CD45^low^CD11b^+^ cells since, the frequency of infiltrating myeloid cells (IMC) is corresponding to CD45^high^CD11b^+^ cells. Furthermore, the frequency of monocytes is corresponding to CD45^high^CD11b^+^LY6C^+^ cells.

### Fluorescence Confocal Microscopy

For tissue fluorescence confocal microscopy, at different time points after surgery, mice were anesthetized, perfused with PBS, and then with 4% paraformaldehyde, sacrificed and the whole brains were obtained. After treatment with sucrose, brain tissues were cut on a freezing sliding microtome at a thickness of 10 μm (Shandom Cryotomo E, Thermo Fisher Scientific). The sections were mounted in adhesive slides (KNITTEL StarFrost^®^ slides, Germany) and were then incubated with 5% normal goat serum (Sigma) in PBS, 0.05% Tween-20 (PBS-T-NGS), for 1 h to reduce nonspecific binding of antibodies to the cell surface and for cell permeabilization. An anti-LC3B (Cell Signaling Technology Beverly, MA, USA) plus anti-LAMP-1 (Abcam, Cambridge, UK) or plus anti-CD45 (Biolegend, San Diego, CA, USA) antibodies were applied to the slides, which were further incubated for 1 h at room temperature. After three rinses with PBS, the slides were incubated with Alexa Fluor 488 plus Alexa Fluor 546 secondary antibodies (Life Technology, Carlsbad, CA, USA) for 60 min. The slides were analyzed under a laser scanning confocal fluorescence microscope (Olympus FV300, Tokyo, Japan). In this experimental procedure, the injection sites were defined as the last section containing a visible needle track and the next section without the needle artifact. Quantification of LC3B^+^ microglial cell numbers and number of vesicles LC3B/LAMP-1^+^ cells in brain slides was performed using the software ImageJ (NIH, USA).

### Detection of Neuronal Degeneration

Neuronal degeneration was analyzed by the Fluoro-Jade B (FJB) techniques. In a first step, slides were staining with anti-NeuN antibody following staining procedures previously described. Briefly, slides were first immersed in a solution of 0.06% potassium permanganate for 10 min. The slides were then rinsed in distilled water for 2 min. The staining solution was prepared from a 0.01% stock solution of FJB (Chemicon) according to the manufacturer’s instructions. To make up staining solution, stock solution was added to 0.1% acetic acid vehicle. This resulted in a final dye concentration of 0.0004% prepared within 10 min of use and was not reused. After 20 min in the staining solution, the slides were rinsed for 1 min in each of three distilled water washes. The slides were then placed on a slide warmer, set at approximately 50°C, until they were fully dry. The dry slides were cleared by immersion in xylene for at least 1 min before coverslipping with DPX (Fluka, Milwaukee, WI, USA; or Sigma Chem. Co., St. Louis, MO, USA). The tissue was then examined using a laser scanning confocal fluorescence microscope (Olympus FV1000, Tokyo, Japan).

### Real-Time PCR

Gene expression for iNOS, IL-1β, IL-6, TNFα, CCR2, and CCL2 were assessed using semiquantitative real-time PCR. Briefly, RNA was isolated from CPU brain cells using a single-step phenol/chloroform extraction procedure (Trizol; Life Technologies, Carlsbad, CA, USA) and depleted of contaminating DNA with RNase-free DNase kit (Life Technologies) before reverse transcription. Reverse transcription was performed on 1 µg of RNA using random hexamers as primers by the high-capacity cDNA RT kit (Life Technologies). Real-time PCR was performed by using One step plus Real-Time PCR System (Applied Biosystems, Foster City, CA, USA). Briefly, 5 ng of reverse-transcribed cDNA was used in triplicate samples. The assays were initiated with 2 min at 50°C, 10 min at 95°C and then 40 cycles of 15 s at 95°C and 1 min at 60°C. Primers were obtained from Applied Biosystems (Foster City, CA, USA). Detection of all target genes and control HPRT was performed using SYBR^®^ Green expression assays Master Mix (Applied Biosystems) and relative quantification (RQ) was calculated by using StepOne™ software V2.2.2 and the RQ = 2^−ΔΔCt^ method, where Ct is the threshold cycle to detect fluorescence. Ct data were normalized to the internal standard HPRT. The primers sequences used are listed in Table 1.

**Table 1:** Real-time PCR primers.

Gene	Real-time PCR primers sequence (5′–3′)
mHPRT1	Sense TCAGTCAACGGGGGACATAAA Antisense GGGGCTGTACTGCTTAACCAG
mCCR2	Sense GTATCCAAGAGCTTGATGAAGGG Antisense GTGTAATGGTGATCATCTTGTTTGGA
mCCL2	Sense CCCACTCACCTGCTGCTACT Antisense TCTGGACCCATTCCTTCTTG
mTNF-α	Sense AGCCGATGGGTTGTACCTTGTCTA Antisense TGAGATAGCAAATCGGCTGACGGT
mIL-1β	Sense ACAGAATATCAACCAACAAGTGATATTCTC Antisense GATTCTTTCCTTTGAGGCCCA
mIL-6	Sense ATCCAGTTGCCTTCTTGGGACTGA Antisense TAAGCCTCCGACTTGTGAAGTGGT
miNOS	Sense GTTCTCAGCCCACAATACAAGA Antisense GTGGACGGGTCGATGTCAC

### Statistical Analysis

All experiments were performed at least three times and the results presented are from representative experiments. Statistical analyses were performed using either Student’s *t*-test for two-group comparison, one-way ANOVA followed by Bonferroni *post hoc* test for more than two groups a one factor or two-way ANOVA followed by Bonferroni *post hoc* test for more than two factors. In all the experiments, *p* < 0.05 was considered to be statistically significant.

## Results

### PGN Injection Induces an Increase in the Frequency of MC in Mouse Brain Parenchyma

**Figure 1:**
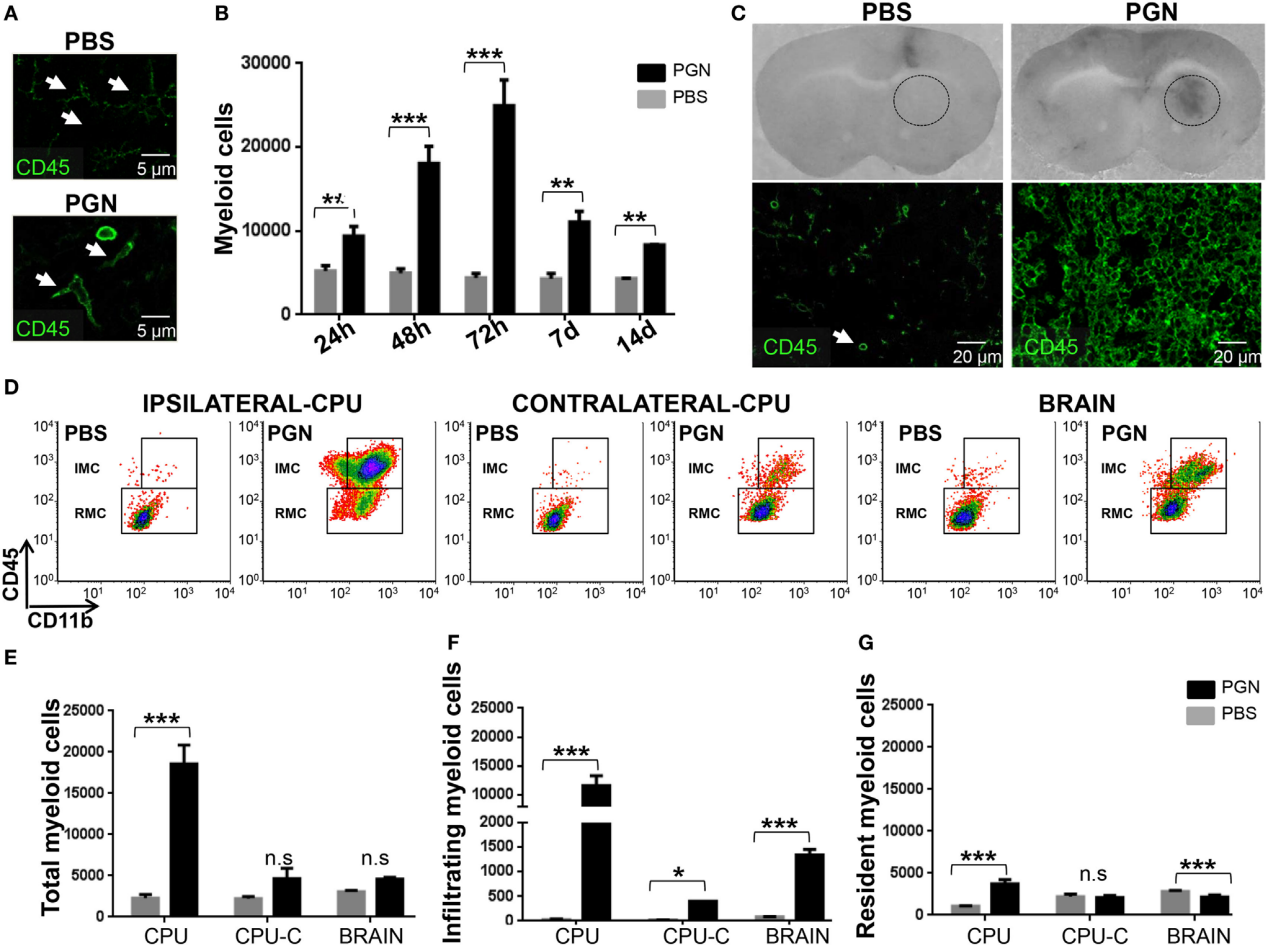
PGN injection induces myeloid cells (MC) recruitment and microglia activation in brain. PGN or PBS was stereotaxically injected into the mouse caudate putamen (CPU). **(A)** At 72 h after injection, 10 µm brain sections were stained with an anti-CD45 (green) antibody. The slides were analyzed under a laser scanning confocal fluorescence microscope. In the PBS-quadrant, arrowheads indicate ramified resting parenchymal microglia, since in the PGN-quadrant, arrowheads indicate activated parenchymal microglial cells. **(B)** At indicated time points after injection, coronal brain slices of 2.0 mm containing the CPU from the injected hemisphere [caudate putamen (CPU)] were dissected. The tissue was homogenized, and CPU cells were costained with anti-CD45 (APC-Cγ7) and anti-CD11b (PERCPE) antibodies and analyzed by flow cytometry gating on CD11b^+^ CD45^+^ MC. The bar graph represents mean ± SD of number of MC from three separated experiments. **(C)** At 72 h after injections, coronal brain slices containing the CPU were photographed. Circle indicate injured injection site. These brain sections were stained with anti-CD45 (green) and then were analyzed under a laser scanning confocal fluorescence microscope, injection site is showed in the picture. **(D)** At 72 h after injection, CPU from injected hemisphere (Ipsilateral-CPU), or CPU of non-injected hemisphere (Contralateral-CPU), or the whole brain (BRAIN) were processed such as indicated in point B. Then, this brain cells were analyzed by flow cytometry gating on CD11b^+^CD45^high^ infiltrating myeloid cells (IMC) and gating on CD11b^+^CD45^low^ resident myeloid cells (RMC). **(E–G)** The bar graph represents mean ± SD of number of MC, of IMC, and of RMC from three separated experiments, respectively. * and *** indicate statistically significant (*p* < 0.05 and *p* < 0.001, respectively) changes compared with PBS-injected mice.

We have previously shown that PGN from *S. aureus* is a potent activator of BV2 microglial line cell (20). To examine *in vivo* if PGN is able to promote activation of resident microglial cells in mouse brain (35), we stereotaxically injected PGN into brain parenchyma (CPU). Microglial cells were analyzed in nervous tissue slices by confocal immunofluorescence microscopy. Staining brain sections with CD45 allowed us to distinguish MC (CD45^+^) from astrocytes, oligodendrocytes, neurons (CD45^−^), and also CD45^bright^ hematogenous population from CD45^dim^ resident microglia. In brains from PBS-injected mice, microglia showed resting morphological features (36, 37), such as radial, non-overlapping processes, a small cell soma and each cell appearing to occupy its own domain (Figure 1A). By contrast, after PGN injection, we observed rounded cells with enlargement of the soma, retraction and shortening of cell processes, resembling amoeboid microglia (Figure 1A). These morphological features are suggesting that PGN induce microglial activation (38), which agrees with our previous *in vitro* data.

Central nervous system damage commonly entails recruitment of circulating immune cells, resulting in an innate immune response that consists of resident microglia and peripherally derived monocytes, macrophages, and dendritic cells (8, 39, 40). Thus, we wanted to determine whether PGN increased MC numbers into the brain parenchyma. After PGN injection, CPU was separated at different time points (24 h, 48 h, 72 h, 7 days, and 14 days) and analyzed for the presence of MC by flow cytometry. As expected, PGN resulted in a significant increase in the number of MC in all time points assessed; reaching a peak of cells recruited 72 h after injection (Figure 1B). These results correlated with the observation by confocal microscopy that the number of CD45^+^ cells, in the vicinity (next three sections without the needle artifact) of the site of injection, increased after the administration of PGN (Figure 1C). Then, to know whether PGN-induced leukocyte recruitment mostly confined to the vicinity of injection, we examined the injection site (ipsilateral-CPU), the opposite non-injected CPU control (contralateral-CPU), and the whole brain (Brain). We identified tissue RMC versus IMC by flow cytometry, using anti-CD45 plus anti-CD11b antibodies (20). PGN injection strongly enhanced the recruitment of inflammatory cells in the CPU, compared with controls (Figures 1D,E). In contralateral-CPU and the whole brain, PGN also increased the number of MC, but in a weaker manner (Figures 1D,E). The increase in the number of MC was mainly due to a rise in the number of CD11b^+^CD45^high^ fraction (IMC) in brains of PGN-injected mice (Figures 1D,F). In addition, we also observed an increase of RMC only confined to ipsilateral-CPU (Figures 1D,G). In agreement with the changes described in the literature for acute injured CNS (1, 41), in these experiments we found that PGN-induced microgliosis, microglial cell activation, and recruitment of IMC in brain tissue.

### Autophagy Requirement for the Recruitment of MC to Brain Parenchyma After PGN Injection

**Figure 2:**
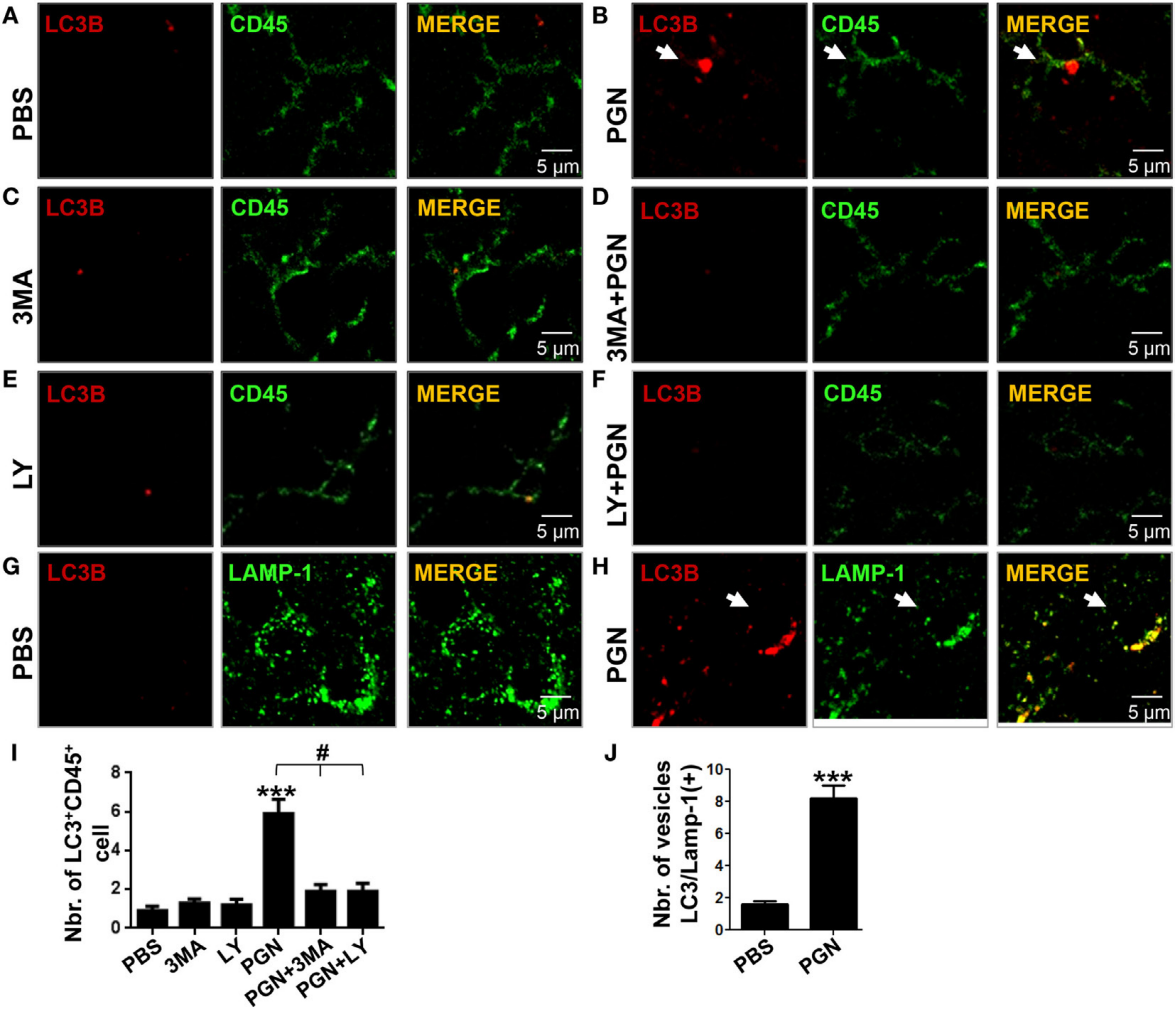
PGN induces myeloid cell autophagy in brain inflammation model. PBS, 3MA, or LY2942002 was stereotaxically injected alone or in combination with PGN into the caudate putamen. **(A–F)** After 24 h, 10 µm brain sections were stained with anti-LC3B (red) plus anti-CD45 (green) or anti-LAMP-1 (green) antibodies. The slides were analyzed under a laser scanning confocal fluorescence microscope. **(A–F)** Arrows indicate the presence of LC3B positive punctuated cells. **(G,H)** Arrowheads indicate colocalization of LC3B positive vesicles with LAMP-1. **(I)** The bars graph represents mean ± SD of number of LC3B^+^ vesicles per CD45^+^ cell of three separated experiments. **(J)** The number of LC3B/Lamp-1 double-positive cells was obtained from 10 fields per slide, analyzing the next three sections without the needle artifact of three separated experiments. *** indicate statistically significant (*p* < 0.001) changes compared with un-stimulated cells.

We previously demonstrated that intracerebral injection of PGN increased autophagy of microglial cells (20). Atg8/LC3 is the most widely monitored autophagy-related protein, and it was originally identified as a subunit of microtubule-associated protein 1 light chain 3 (42). The induction of autophagy in PGN-injected mice was monitored by morphometric analysis after the formation of LC3B-labeled autophagosomes (≥1 μm) (43, 44). In this study, we confirmed that PGN induced an increase in the number of LC3B^+^ puncta in CD45^+^ cells, in the brain parenchyma, compared with control (Figures 2A,B). In addition, PI3K inhibitors, such as LY294002 and 3-MA, that block class I as well as class III PI3Ks, prevent autophagosome formation, and finally suppress autophagy (23, 45), were able to prevent the increase of LC3B punctate parenchymal MC by PGN treatment (Figures 2C–F,I). Moreover, we next examined whether PGN induces convergence of the autophagic pathway with a functional degradative compartment. Staining brain sections with antibodies against LC3B plus the LAMP1 allowed us to visualize the fusion of autophagosomes with lysosomes by confocal microscopy (46, 47). Similarly, to our previous report (20), PGN injection into the CPU significantly induced overlapping signals of LC3B and LAMP1 compared with control, suggesting the autophagic flux is taking place (Figures 2G,H,J).

**Figure 3:**
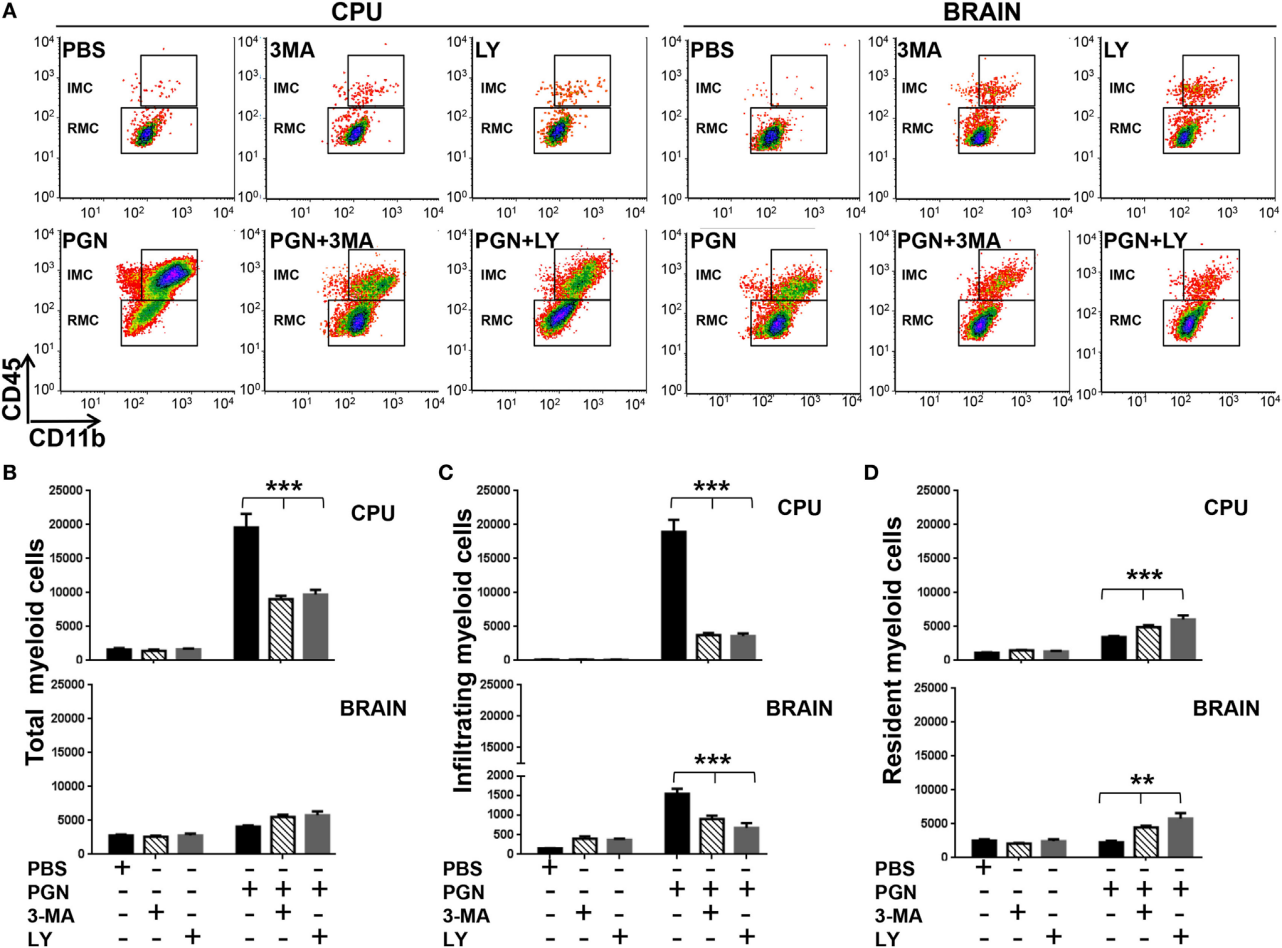
PGN-induced myeloid cell recruitment requires PI3K. PGN or PBS was injected with 3-methyladenine (3-MA) or LY2942002 into the caudate putamen (CPU). **(A)** At 72 h after injection, CPU or BRAIN cells were analyzed by flow cytometry gating on MC. **(B–D)** The bar graph represents mean ± SD of number of MC, of infiltrating myeloid cells and of resident myeloid cells from three separated experiments. * and *** indicate statistically significant (*p* < 0.05 and *p* < 0.001, respectively) changes PGN + 3-MA or PGN + LY2942002 compared with PGN-injected mice.

Next, we determined whether the presence of 3-MA and LY294002 affected inflammatory recruitment in the CPU of PGN-injected mice. Wild-type (WT) mice were injected with PGN plus 3-MA or PGN plus LY294002, 72 h later, the number of MC was analyzed by flow cytometry. Interestingly, we found that both autophagy inhibitors, 3-MA or LY294002, decreased the number of MC in the CPU of PGN-injected mice (Figure 3A), and this effect was mostly confined to MC in the ipsilateral side, since the analysis of the whole brain, showed that both inhibitors failed to modify the number of cell populations (Figure 3B). In addition, here we reveal that these autophagy inhibitors decreased the number of IMC cells in the PGN-injected parenchymal mice but increased the number of RMC (Figures 3C,D). Similar effects of these inhibitors were observed at the whole brain (Figures 3C,D). Taking into consideration that LY294002 and 3-MA inhibited the response to PGN and these molecules can inhibit autophagy, these results suggest that PI3K and autophagy response may be required for the MC recruitment in PGN-injected brains.

### TLR2 and MyD88 Signaling Regulates PGN-Induced Leukocyte Entry to the CNS Parenchyma

**Figure 4:**
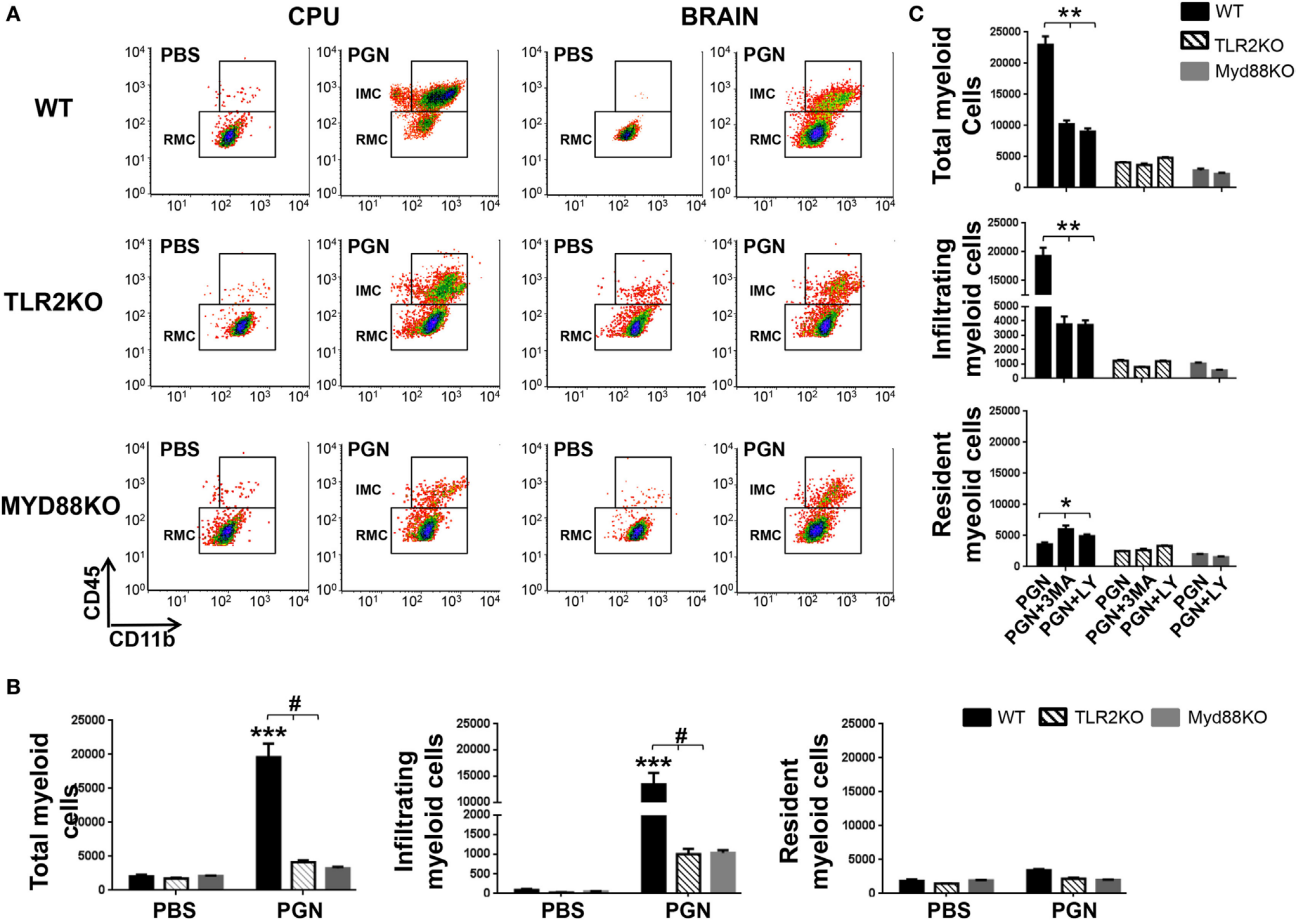
PGN-induced myeloid cell recruitment requires MyD88/TLR2 signaling. **(A)** PGN or PBS was injected into the caudate putamen (CPU) from MyD88 KO, TLR2KO, or C57BL/6 mice. At 72 h after injection, CPU or BRAIN cells were analyzed by flow cytometry gating on MC. **(B)** The bar graph represents mean ± SD of number of MC, of infiltrating myeloid cells (IMC) and of resident myeloid cells (RMC) from three separated experiments. * and *** indicate statistically significant (*p* < 0.05 and *p* < 0.001, respectively) changes PGN-injected TLR2 KO mice or PGN-injected MyD88 KO mice compared with PGN-injected wild-type (WT) mice. **(C)** The bar graph represents mean ± SD of number of MC, of IMC, and of RMC from three separated experiments. * and *** indicate statistically significant (*p* < 0.05 and *p* < 0.001, respectively) changes PGN + 3-methyladenine (3-MA) or PGN + LY2942002 injected WT mice compared with PGN-injected WT mice.

To confirm the involvement of TLR2 and MyD88 adaptor protein in the recruitment effects of PGN, we studied leukocyte recruitment to mouse brain parenchyma after PGN intracerebral injection in TLR2KO and MyD88KO mice. We found that recruitment of MC in parenchymal CNS was significantly lower in TLR2KO mice compared with the WT mice, 72 h after PGN injection (Figures 4A,B). Consistent with this result, we observed that PGN injection in TLR2KO mice failed to increase the number of IMC, RMC in the ipsilateral-CPU and in whole brain, compared with the PGN-injected WT mice (Figures 4A,B). Similar results were obtained in MyD88KO mice, since we did not observe the major MC recruitment in the ipsilateral-CPU and whole brain of MyD88KO mice after PGN injection (Figures 4A,B). Taken together, our experiments demonstrate that the activation of TLR2 by PGN and signaling through the MyD88 adaptor protein are an important regulator of leukocyte recruitment after PGN injection in the brain parenchyma.

As showed above, PI3K inhibitors regulate MC entry to PGN-injected CPU. As expected, in additional experiments, we observed that coinjection of PGN plus 3-MA or PGN plus LY294002 in TLR2KO and MyD88KO mice, did not reveal significantly differences in the leukocyte recruitment in the ipsilateral-CPU and whole brain of these mice compared with the controls (Figure 4C). Thus, this experiment suggests that PI3K inhibitors does not affect leukocyte entry to CNS parenchyma, in the absence of TLR2/MyD88-mediated PGN signaling.

### PI3K Inhibitors Modulate Inflammatory Cell Phenotype and Pro-Inflammatory Mediators in PGN-Injected CPU

**Figure 5:**
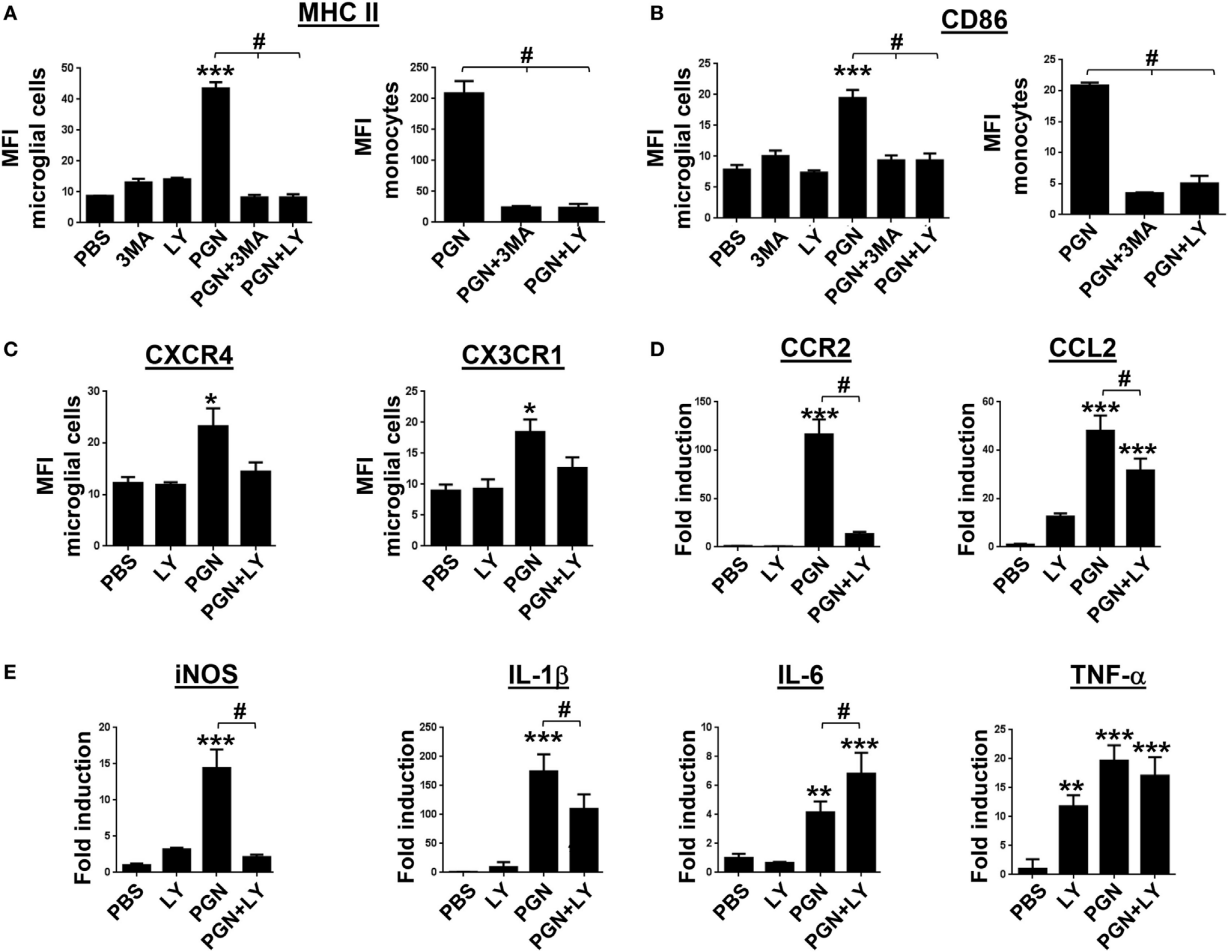
Inhibition of PI3K modulates PGN-induced pro-inflammatory myeloid cells activation. PGN or PBS was injected with 3-methyladenine (3-MA) or LY2942002 into the caudate putamen (CPU). **(A–C)** At 72 h after injection, CPU cells were stained with anti-IA/IE, anti-CD86, anti CXCR4, and anti-CX3CR1 antibodies and analyzed by flow cytometry gating on microglial cells (CD11b^+^CD45^low^ cells) or monocytes (CD11b^+^CD45^high^Ly6C^+^). The bar graph represents mean ± SD of mean fluorescence intensity (MFI) from three separated experiments. * and *** indicate statistically significant (*p* < 0.05 and *p* < 0.001, respectively) changes PGN compared with PBS-injected mice, ^#^ indicate statistically significant (*p* < 0.05 and *p* < 0.001, respectively) changes PGN + 3MA or PGN + LY2942002 compared with PGN-injected mice. **(D,E)** At 72 h after intracerebral injection, total RNA was extracted from CPU tissues and examined for mCCR2, mCCL2 miNOS, mIL-1β, mIL-6, and mTNF-α genes expression by real-time PCR. Arbitrary units were used to indicate the fold difference in PGN versus PBS-injected mice after normalization with the HPRT transcripts. ^#^ indicate the fold difference in PGN + LY2942002 versus PGN-injected mice.

Expression of MHC class II and CD80 and CD86 costimulatory molecules is associated with the ability to present antigen, and their detection can be used to indicate MC activation (48). Previous studies demonstrated that LPS (TLR4 ligand) was able to increase MHC class II, CD80, and CD86 expression in primary microglia isolated from human adult patients (49). However, there is little evidence in the literature if another TLR family members, such as TLR2, could regulate activation of microglia, within damaged CNS. Here, we explored the expression of molecular markers related to the cell activation, in MC from PGN-injected mice, in the presence/absence of autophagy inhibitors. We found that PGN injection increased expression of MHC class II and CD86 in microglial cells and monocytes. Interestingly, 3-MA and LY294002 treatment prevented the effects of PGN on the expression of both molecular markers in these cell types (Figures 5A,B). Chemokines and their receptors are expressed in the CNS, where they play key functions in development and maintenance (50, 51). For instance, CX3CL1/CX3CR1 signaling modulates stimulus-dependent microglial activation (52). Moreover, CXCR4 is upregulated in microglia and astrocytes in various brain diseases, such as HIV encephalitis and experimental allergic encephalomyelitis (53, 54). We investigated the expression of CXCR4 and CX3CR1 in microglia and infiltrating monocytes after PGN intracerebral injection. We observed that PGN increased CXCR4 and CX3CR1 expression in microglia compared to the control, and their expression was significantly inhibited in the presence of LY294002 (Figure 5C). However, this inhibitor did not affect the chemokines expression in monocytes from PGN-injected mice (data not shown). In addition, we investigated whether CCL2 and CCR2 are increased in PGN-injected mice compared with controls. This signaling axis is important on monocyte recruitment to CNS tissue during immune-mediated inflammation (55). We detected increased CCL2/CCR2 gene expression in PGN-injected CPU, compared with controls (Figure 5D), which was prevented by LY294002 injection (Figure 5D). Microglia activation is associated with the secretion of cytokines that later can modulate their activation state. High levels of pro-inflammatory cytokines such as TNF-α, IL-6, and IL-1β were found elevated in the brain tissue from patients with neurological diseases (56). It has also been reported that PGN increases iNOS and COX-2 expression in BV2 microglial cells by binding to the TLR2 receptor/MyD88 which in turn activates PI3K/AKT/NF-kappa B signaling pathway (57). Here, we observed that PGN injection promoted iNOS, IL-1β, IL-6, and TNF-α gene expression compared with controls (Figure 5E). In addition, we studied if PI3K inhibition modulates pro-inflammatory cytokines expression after PGN treatment. We found that coinjection LY294002 with PGN into CPU, prevented PGN-induced iNOS and IL-1β production (Figure 5E), but PGN-induced IL-6 production was increased in presence of this inhibitor (Figure 5E). Moreover, LY294002 was not able to change PGN-induced TNF-α production (Figure 5E). These results suggest that PI3K inhibitors could selectively regulate iNOS and IL-1β production, in addition to leukocyte recruitment, in PGN-injected mice.

### PI3K Inhibitors Prevent PGN-Induced Neuronal Cell Death

**Figure 6:**
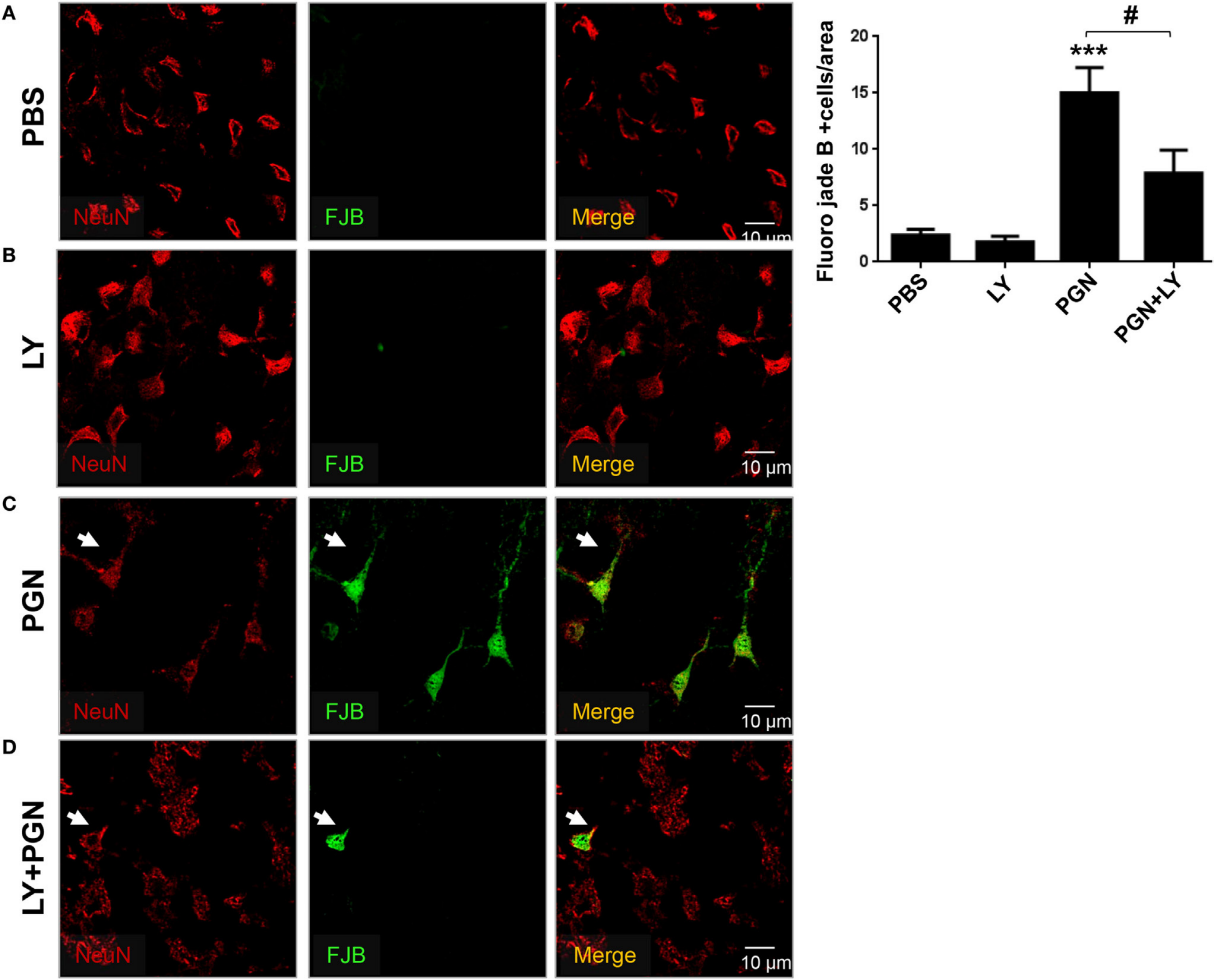
Pharmacological inhibition of PI3K prevents neuronal loss in PGN-injected brain. PGN or PBS was injected with LY2942002 into the caudate putamen. **(A–D)** At 72 h after injection, 20 µm brain sections were stained with anti-NeuN (red) antibody. Then, the slides were stained with Fluor-Jade B (FJB) work solution and analyzed under a laser scanning confocal fluorescence microscope. Arrowheads indicate the presence of FJB positive neuron cells. The bars graph represents mean ± SD of number of FJB + neuron cells from three separated experiments. *** indicate statistically significant (*p* < 0.001) changes PGN compared with PBS-injected mice.

Finally, we examined if PGN was able to induce neuronal damage in mouse brain, using FJB staining and confocal microscopy. We detected neuronal toxicity generation in CPU sections of PGN-injected mice compared with the controls (Figures 6A,C). Furthermore, we observed that the coinjection of LY294002 reduced neuronal cell death induced by PGN (Figures 6B–D). Therefore, these data suggest that PI3K signaling regulate neuronal damage induced by PGN injection.

## Discussion

Recent studies have provided key insights into TLR-mediated activation of PI3K, suggesting that the outcome of PI3K activation downstream of these immune receptors depends on both the TLR that is stimulated and the cell type being activated (34). In this study, we have uncovered a completely novel role for PI3K in the control of brain inflammation. We have shown that the PGN injection into the brain parenchyma triggered LC3B^+^ puncta in CD45^+^ cells, LC3B and LAMP1 colocalization, recruitment of MC to the injection site, pro-inflammatory mediators’ production, and neurodegeneration. Moreover, these effects of PGN were prevented by PI3K inhibitors (including 3-MA). To the best of our knowledge, this is the first demonstration that class III and class I PI3K inhibitors, that may also modulate autophagy, a fundamental homeostatic cell process, could control inflammatory response in the CNS after TLR2 stimulation.

Myeloid cells in CNS represent a heterogeneous class of innate immune cells that contribute to the maintenance of tissue homeostasis differentially during development and adulthood (58, 59). Microglia are the only MC type in the CNS parenchyma under the steady-state conditions and act as the first line of defense in the nervous system (8). The morphologically “resting” microglia are continually surveying their microenvironment with their highly motile processes. Upon detection of focal injury to the brain, microglial processes rapidly move toward the lesion site, and switching their behavior, leading to focal microglial activation (35). This work provides evidence that PGN-induced focal activation of microglia confined to the CPU. We observed microglial cell accumulation with morphology distinguishable from the resident microglial cells, consisting of shorter, asymmetrically oriented processes as well as enlarged cell bodies nearly to the injection site. In addition, we demonstrated that PGN present in the mouse brains not only induced microgliosis but also augmented other CNS-infiltrating MC populations in the injected site. In line with these findings, other authors previously reported that PGN in the brain of multiple sclerosis patients could contribute to the disease progression through the activation of infiltrating dendritic cells (60). Recently, Luz et al. found that TLR2 activated in CNS affects the innate but not adaptive brain immune responses. Our findings revealed that stimulation of TLR2 in brain parenchyma (CPU) triggers MC recruitment in the several areas analyzed (61). We observed that PGN increased MC numbers in CNS 72 h after injection, but this response was controlled 14 days after treatment. This evidence suggests that the parenchymal PGN injection could temporally activate CNS MC, which mediate local inflammation and later these cell populations return to steady state.

Under normal conditions, microglial cells contribute to the restriction of other MC to access the CNS parenchyma. However, neuroinflammatory circumstances, such as neural injury or local inflammation, trigger defined molecular mechanisms that allow other non-parenchymal mononuclear phagocytes (those found in the choroid plexus, meninges, and perivascular spaces) and peripheral immune cells to breach the glia limitans and enter the CNS parenchyma (62, 63). We observed that PGN injection into brain parenchyma lead to the migration of MC, which were mostly confined around the injection site. Interestingly, two PI3K inhibitors, such as 3-MA and LY294002, were able to block this MC recruitment. Taking into consideration that 3-MA prevent autophagosome formation, these results indicate that microgliosis and recruitment of others MC induced by TLR2 activation may require autophagy activation. These results are in agreement with a recent publication showing that genetic inhibition of autophagy prevented irradiation-induced microglial activation and neuroinflammation (64). On the other hand, Jin et al. found that intracerebral administration of the autophagy inhibitor 3-MA promoted microglia activation after traumatic brain injury (65). Taking into consideration the results of the present study and other reports, we propose that autophagy may play different roles in modulation of MC activation and inflammation, depending on the inflammatory context and other microenvironment factors. For instance, basal levels of autophagy are required for normal neuron survival (28, 66), while overactivated autophagy induces autophagic cell death (67).

Although no solid evidence about the role of MC autophagy on brain inflammation has emerged, recent research showed that neuronal TLR2 activation has been associated with the increased levels of the autophagy/lysosomal pathway marker p62 as well as with the recruitment and activation of microglia in the substantia nigra of PD brain (68). In this sense, autophagy is regulated by a plethora of immunological signals, including ligands for pattern-recognition receptors, for example, TLRs and cytokines (69, 70). In this article, we showed that PGN, a TLR2 ligand, triggers autophagy activation in brain MC and autophagolysosome formation, and this effect was blocked by PI3K inhibitors. Considering all these results, we explored the role of TLR2 signaling in PGN-induced brain inflammation. We observed that TLR2 and the common TLR signaling adaptor MyD88 were required for the PGN-induced MC recruitment and MyD88 and TLR2 deficiency mainly affected infiltrating MC accumulation instead resident MC. In accordance with our results, it was described in the literature that MyD88/TLR2 signaling regulated infiltration of the peripheral immune cells populations and microglial expansion in response to the acute brain inflammation (71, 72). Thus, we confirm that MyD88/TLR2 signaling plays a pivotal role in PGN-induced inflammatory response and this effect may involve PI3K and autophagy activation in CNS MC.

Several pro-inflammatory factors produced by CNS MC also influence the evolution of neuroinflammatory injury (73). Previous studies have shown that inflammation leads to the accumulation of perivascular macrophages and microglial cells at the inflammatory site, and they upregulate MHC class II expression and costimulatory molecules (74). In agreement with these studies, we also revealed that PGN injection increased MHC-II and CD86 expression in microglia and monocytes at the inflammatory site and PI3K inhibition reduced these PGN effects. These findings are in agreement with the concept that MC in the CNS are highly specialized but also plastic cells, that become reactive in the context of any changes in CNS homeostasis. In this sense, we suggest that the PI3K pathway participate in determining the phenotypic profile of MC and its inhibition could lead to limitation of CNS inflammation.

Multiple mechanisms could account for the induction in MC recruitment observed in PGN-injected mice. Based on previous reports describing that fractalkine/CX3CR1 signaling regulates microglial behavior in several CNS disorders (75), we evaluated the CX3CR1 expression in microglial cells after PGN treatment. Interestingly, since we observed that PGN increased CX3CR1 expression in these cells, LY294200 was able to attenuate this PGN-effect. In addition, we found that PGN also increased CXCR4 expression in microglial cells, and this upregulation was diminished by PI3K inhibition. These findings suggest that inhibition of PI3K could regulate inflammatory response in CNS from PGN-injected mice involving mechanisms that reduce migration of peripheral MC to injury site and modulate phenotype profile of resident MC.

In pathological conditions, microglia can activate endothelial cells by the release of reactive oxygen species and cytokines such as IL-1α, IL-1β, IL-6, and TNF-α (76, 77). Also, the recruitment of peripheral immune cells can be initiated by microglia through the secretion of the chemokine CCL2 under inflammatory conditions (78). Here, we demonstrated that PGN promoted and increase in iNOS, IL-1β, IL-6, and TNF-α gene expression in cells from the injection site. Moreover, PI3K inhibition differentially modulated gene expression of these mediators. Finally, in this set of experiments, we observed an increase in CCR2 and CCL2 gene expression in CPU from the PGN-injected mice compared with the control animals. Interestingly, LY294200 diminished gene expression of both molecules studied.

There are different glial cell populations (astrocytes and microglial cells) and infiltrating MC, responsible for the release of inflammatory mediators in the CPU from PGN-injected mice. Therefore, we suggest that the global effect of PI3K inhibition of inflammatory mediator expression may be affected by the presence of different cell types, that may produce distinct mediators, since PI3K signaling modify cell recruitment.

Brain inflammation is a typical feature of neurodegenerative diseases (79–81) and could be prominent sequel of many acute forms of brain injury (for example, trauma, encephalitis, and stroke) (82, 83). Under certain circumstances, neuroinflammation is known to promote neuronal death (84). Our findings suggest that PGN induced neuronal cell death involving autophagy activation. Indeed, our studies have shown that MC exposed to PGN failed to be toxic to neighboring neurons in the presence of LY294200.

In this sense, was reported that autophagy activation in cerebral ischemia has a destructive role but this effect was prevented by the administration of 3-MA (85, 86). Conversely, other evidences have suggested that autophagy has a neuroprotective function (87, 88). We support the idea that physiological levels of autophagy are favorable to neuronal survival, but excessive or inadequate levels, could be harmful and cause injury.

To summarize, this study suggests a new role for PI3Ks and PGN-induced autophagy, in MC recruitment to the CNS and brain inflammation. Considering that recent discoveries point to autophagy as a substantial regulator of CNS innate immune responses and our findings that pharmacological inhibitors of class I and class III PI3K can suppress both, the inflammatory response and neuronal toxicity, we proposed that these molecules could potentially be consider as targets for exploration and development of new therapeutic strategies for neurodegenerative diseases.

## Ethics Statement

All experiments were done in compliance with the procedures outlined in the “Guide for the Care and Use of Laboratory Animals” (NIH Publication No. 86-23, 1985). The experimental protocols were approved by the Institutional Animal Care and Use Committee (IACUC). Our animal facility obtained NIH animal welfare assurance (No. A5802-01, OLAW, NIH, USA).

## Author Contributions

DA designed and carried out all the experiments and wrote the manuscript. EG helped in setting up *in vivo* experiments and confocal microscopy studies. JR and CB helped to perform *in vivo* studies. MA and LC helped in setting up and carried out intracerebral injections. PI conceived and designed the study, supervised all of the experiments, and helped to write the manuscript. All of the authors discussed the results and commented on the manuscript.

## Conflict of Interest Statement

The authors declare that the research was conducted in the absence of any commercial or financial relationships that could be construed as a potential conflict of interest.
